# Factors for success of awake prone positioning in patients with COVID-19-induced acute hypoxemic respiratory failure: analysis of a randomized controlled trial

**DOI:** 10.1186/s13054-022-03950-0

**Published:** 2022-03-28

**Authors:** Miguel Ibarra-Estrada, Jie Li, Ivan Pavlov, Yonatan Perez, Oriol Roca, Elsa Tavernier, Bairbre McNicholas, David Vines, Miguel Marín-Rosales, Alexandra Vargas-Obieta, Roxana García-Salcido, Sara A. Aguirre-Díaz, José A. López-Pulgarín, Quetzalcóatl Chávez-Peña, Julio C. Mijangos-Méndez, Guadalupe Aguirre-Avalos, Stephan Ehrmann, John G. Laffey

**Affiliations:** 1 Unidad de Terapia Intensiva, Hospital Civil Fray Antonio Alcalde, El Retiro, Coronel Calderón 777, Guadalajara, Jalisco Mexico; 2 Department of Cardiopulmonary Sciences, Division of Respiratory Care, Rush University, Chicago, IL USA; 3 Department of Emergency Medicine, Hôpital de Verdun, Montréal, Québec Canada; 4 CHRU Tours, Médecine Intensive Réanimation, CIC INSERM 1415, CRICS-TriggerSep FCRIN Research Network, Tours, France; 5 Servei de Medicina Intensiva, Hospital Universitari Vall d’Hebron, Barcelona, Spain; 6 Ciber Enfermedades Respiratorias (CIBERES), Instituto de Salud Carlos III, Madrid, Spain; 7 Clinical Investigation Center, INSERM 1415, CHRU Tours, Tours, France; 8 Methods in Patients-Centered Outcomes and Health Research, INSERM, UMR 1246, Nantes, France; 9 Department of Anaesthesia and Intensive Care Medicine, Galway University Hospitals, National University of Ireland, Galway, Ireland; 10 HRB Galway Clinical Research Facility, and School of Medicine, National University of Ireland, Galway, Ireland; 11 Servicio de Medicina Interna, Hospital General de Occidente, Guadalajara, Jalisco Mexico; 12 Departamento de Urgencias, Hospital Civil Fray Antonio Alcalde, Guadalajara, Jalisco Mexico; 13 Departamento de Enfermedades Infecciosas, Hospital Civil Fray Antonio Alcalde, Guadalajara, Jalisco Mexico; 14 INSERM, Centre d’étude des pathologies respiratoires, U1100, Université de Tours, Tours, France

**Keywords:** Awake prone positioning, COVID-19, Acute hypoxemic respiratory failure, Intubation

## Abstract

**Background:**

Awake prone positioning (APP) improves oxygenation in coronavirus disease (COVID-19) patients and, when successful, may decrease the risk of intubation. However, factors associated with APP success remain unknown. In this secondary analysis, we aimed to assess whether APP can reduce intubation rate in patients with COVID-19 and to focus on the factors associated with success.

**Methods:**

In this multicenter randomized controlled trial, conducted in three high-acuity units, we randomly assigned patients with COVID-19-induced acute hypoxemic respiratory failure (AHRF) requiring high-flow nasal cannula (HFNC) oxygen to APP or standard care. Primary outcome was intubation rate at 28 days. Multivariate analyses were performed to identify the predictors associated to treatment success (survival without intubation).

**Results:**

Among 430 patients randomized, 216 were assigned to APP and 214 to standard care. The APP group had a lower intubation rate (30% vs 43%, relative risk [RR] 0.70; CI_95_ 0.54–0.90, *P* = 0.006) and shorter hospital length of stay (11 interquartile range [IQR, 9–14] vs 13 [IQR, 10–17] days, *P* = 0.001). A respiratory rate ≤ 25 bpm at enrollment, an increase in ROX index > 1.25 after first APP session, APP duration > 8 h/day, and a decrease in lung ultrasound score ≥ 2 within the first 3 days were significantly associated with treatment success for APP.

**Conclusion:**

In patients with COVID-19-induced AHRF treated by HFNC, APP reduced intubation rate and improved treatment success. A longer APP duration is associated with APP success, while the increase in ROX index and decrease in lung ultrasound score after APP can also help identify patients most likely to benefit.

*Trial registration*: This study was retrospectively registered in ClinicalTrials.gov at July 20, 2021. Identification number NCT04477655. https://clinicaltrials.gov/ct2/show/NCT04477655?term=PRO-CARF&draw=2&rank=1

**Supplementary Information:**

The online version contains supplementary material available at 10.1186/s13054-022-03950-0.

## Background

A significant proportion of patients with COVID-19 require hospitalization and oxygen support due to progressive respiratory failure [1]. Intubation rate varies from 15 to 85% [2] and is a major concern in the regions with the highest mortality [3–5] due to the unprecedented pressure on healthcare systems. Prone positioning is standard care for intubated patients with moderate to severe acute respiratory distress syndrome (ARDS) [6, 7], with the physiological benefits of improving lung heterogeneity and aeration, which ultimately lead to improved survival.

Considering the potentially similar physiological mechanism, awake prone positioning (APP) has been broadly applied for non-intubated patients with COVID-19 since the early pandemic. However, despite the wide adoption of this maneuver and its recommendation in the updated National Institutes of Health Guidelines [8], there is scarce evidence regarding early predictors of success and sufficient monitoring of clinical response after initiation of APP; such data might help select the patients who will benefit the most, and early identify those with inadequate response, avoiding unnecessary delay in escalation of care.

We designed this multicenter open-label randomized controlled trial (NCT04477655) to assess the potential for APP [9], as compared to standard care, to reduce the need for intubation and invasive ventilation of patients with COVID-19-induced acute hypoxemic respiratory failure (AHRF). Shortly after initiation of the trial, we joined a consortium of five other national multicenter trials to establish a collaborative prospective meta-trial, with the advantage of more rapidly determining the effectiveness of APP in patients with severe COVID-19, a key priority given the pandemic situation [10, 11]. This consortium recently published the strongest available evidence in favor of APP [12], but only the outcomes that were common across all trials were reported, including baseline characteristics, APP duration, and outcomes (28-day intubation rate and mortality). As prospectively defined, this paper reports additional data prospectively collected in our trial that were not reported in the meta-trial publication [12] and are important to understanding how to maximize the therapeutic potential of APP by identifying the factors associated with APP success [10].

## Materials and methods

### Trial design and oversight

We conducted a parallel randomized controlled trial at two university hospitals in western Mexico, in which three high-acuity units dedicated to treat COVID-19 patients were included, with patient-to-nurse ratio of 4:1 in two units and 2:1 in one unit. The protocol was approved by the ethic committees at both hospitals. This trial was registered at clinicaltrials.gov with the identification number NCT04477655.

### Patients

Patients aged ≥ 18 years with reverse-transcriptase polymerase chain reaction (RT-PCR) confirmed COVID-19, and pulse oximetry (SpO_2_) < 90% despite receiving oxygen at 15 L/min through a non-rebreather mask, were initiated on high-flow nasal cannula (HFNC) and assessed for eligibility. Inclusion criteria were the requirement of a fraction of inspired oxygen (F_I_O_2_) ≥ 0.3 through HFNC at the maximum tolerated flow to maintain a SpO_2_ ≥ 90%. Exclusion criteria included severe respiratory failure requiring immediate intubation, do-not-intubate/resuscitate orders, laparotomy within 2 weeks, pregnancy, vasopressor requirement to maintain median arterial pressure > 65 mmHg, and refusal to participate.

For all COVID-19 patients admitted to our hospitals, silent hypoxemia was assessed before initiating oxygen therapy and documented by clinicians at hospital admission using a prespecified definition of SpO_2_ < 90% at ambient air without perception of dyspnea or shortness of breath [13, 14].

### Randomization and intervention

After signing informed consent, patients were randomly assigned to receive standard care or APP using a predetermined randomization sequence prepared in sealed opaque envelopes. The sequence was generated by computer and stratified by center, with a 1:1 allocation ratio, using permuted blocks with a size of 4. Due to the nature of the intervention, blinding was impossible.

HFNC was initiated at maximal flow of 40 L/min (Vapotherm, Precision flow, Exeter, NH, USA), with F_I_O_2_ titrated to maintain SpO_2_ between 92% and 95%. Flow and temperature were then titrated based on patient comfort. HFNC was maintained until patients met weaning criteria, defined as a requirement of a F_I_O_2_ ≤ 0.4 and flow ≤ 20 L/min to maintain SpO_2_ ≥ 90% for ≥ 2 h; death; need for noninvasive ventilation (NIV) or intubation.

Study investigators maintained daily communication with on-site clinicians minimally three times/day to emphasize the protocol adherence. Labels indicating the allocation arm were placed inside patient rooms to remind the patients to stay in the assigned group. Patients in the APP group were consistently encouraged by the bedside clinicians to remain in APP as long as possible, and assistance to reposition was offered as needed, with pillows placed at chest, pelvis, and/or knees to improve comfort. Personal cell phone use with Internet connection was encouraged, in order to distract patient attention and maximize their tolerance for APP.

During the first APP session, patient’s physiological variables pre-APP, 1 h during APP, and 1 h after returning to supine position were recorded, including respiratory rate, SpO_2_/F_I_O_2_, and the ratio of SpO_2_/F_I_O_2_ to respiratory rate, known as the ROX index [15]. The APP duration in each session was recorded by nurses. Patients who received APP < 1 h/day during HFNC were excluded from the *per*-protocol analysis.

In the standard care group, APP was discouraged. If APP was performed for ≥ 1 h, patients were excluded from the *per*-protocol analysis.

Based on early evidence of prognostic role of D-dimer levels, they were prospectively recorded for all patients at enrollment [16].

Attending physicians, who were all certified trainers of WINFOCUS (*World Interactive Network Focused on Critical Ultrasound*, https://www.winfocus.org/), recorded daily lung ultrasound (LUS) score in all enrolled patients under supine position from study enrollment and until the third day. The LUS score was interpreted and recorded by the same clinicians, based on the lung ultrasound pattern in the ventral, lateral, and dorsal sections of both lungs (12 sections), with maximal score of 3 in each section and 36 in total, higher score indicating lower aeration [17, 18].

### Outcomes

All patients were followed up until 28 days of enrollment, with intubation as the primary outcome. Intubation criteria were predetermined: respiratory rate ≥ 40 breaths/min, respiratory muscle fatigue, respiratory acidosis with pH ≤ 7.25, copious tracheal secretions, SpO_2_ < 80% despite an F_I_O_2_ of 1.0, hemodynamic instability, anxiety due to dyspnea, or deteriorating mental status.

Secondary outcomes included treatment success, which was defined as being alive without intubation at day 28, mortality at 28 days, HFNC duration, use of NIV, time to intubation, days of invasive ventilation, hospital length of stay, physiological response to the first APP session, and adverse events.

Exploratory outcomes included the predictors for treatment success in the overall population and in the APP group, the association between treatment success and mean daily duration of APP, and the difference in outcomes among patients with silent hypoxemia.

### Sample size and statistical analysis

Assuming an intubation rate of 38%, the calculated sample size was 468 patients (234 per group) in order to detect an absolute difference of 12% to provide a statistical power of 80% and *α* = 0.05.

The predictors for treatment success in the overall population and in the APP group were assessed with multivariate logistic regression analysis, using the enter method. A linear relationship was investigated between probability of success and the duration of APP within the first 3 days, and it was plotted in a scatter diagram. Receiver operating characteristic (ROC) curve analysis was performed for duration of APP and other continuous predictive variables, using treatment success as the end point; Kaplan–Meier curves were plotted for treatment success and death between patients above and under the best cutoff according to the Youden index (8 h) and were compared with the log-rank test and adjusted with a Cox proportional hazards regression. We performed a subgroup analysis of the outcomes in patients with silent hypoxemia at hospital admission. A two-tailed *P* value of less than 0.05 was considered as statistically significant. We used SPSS software, version 27 (IBM), and GraphPad Prism software (version 9) for all the analyses and graphics.

## Results

**Fig. 1:**
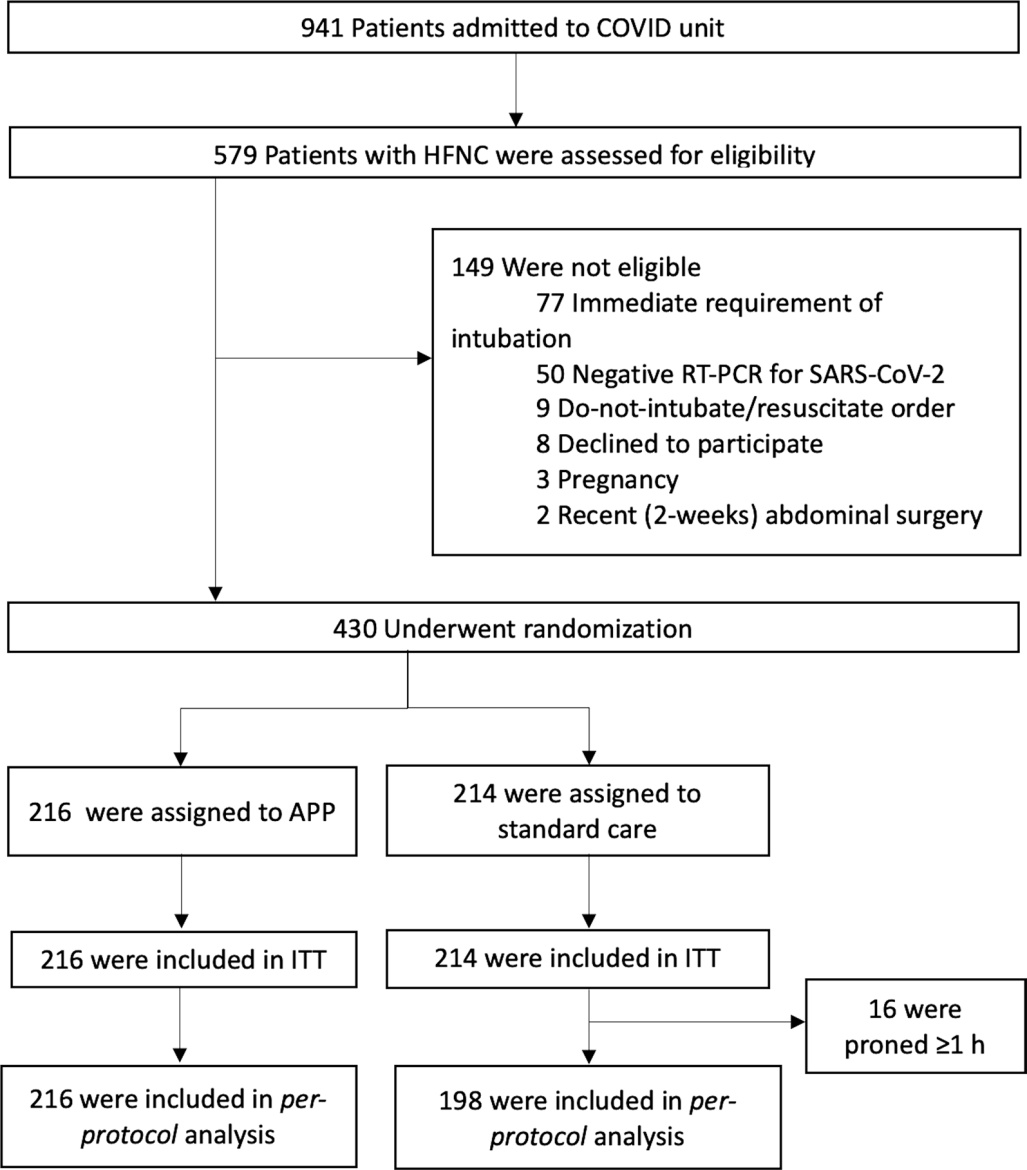
Flowchart of participants. HFNC, high-flow nasal cannula; RT-PCR, reverse-transcription polymerase chain reaction; APP, awake prone positioning; ITT, intention to treat

From May 5, 2020, to January 26, 2021, 941 patients with COVID-19-induced AHRF were admitted; 579 patients were assessed for eligibility, of whom 430 underwent randomization (Fig. 1), with 216 patients assigned to APP and 214 to standard care. This trial was interrupted before reaching planned enrollment (*n* = 468) based on an interim analysis at the meta-trial level, demonstrating APP superiority over standard care [12].

Patients in APP and standard care groups had similar characteristics at study enrollment (Table 1). Silent hypoxemia was observed at hospital admission in 117 patients (27%), with a similar proportion in APP and control groups. This subgroup had a lower proportion of female patients, a lower respiratory rate, and lung ultrasound score at enrollment when compared to the 313 patients with dyspneic hypoxemia, despite having similar SpO_2_/F_I_O_2_ ratio and comorbidities (Table 2).

**Table 1:** Baseline characteristics and outcomes according to allocated group

	APP (n = 216)	Standard care (n = 214)	RR (95% CI)	*P*
*Characteristics*				
Age—years	58.6 ± 15.8	58.2 ± 15.8	–	–
Female sex—no. (%)	84 (38.9)	88 (41.1)	–	–
Body mass index^a^—kg/m^2^	30.3 ± 4.6	30.0 ± 3.8	–	–
Days from symptoms onset to hospital admission	8 (7–10)	8 (7–9)	–	–
SpO_2_ at hospital admission—%	79 (75–84)	80 (75–85)	–	–
Hours from admission in hospital to study enrollment	17 ± 9.3	16 ± 9.9	–	–
Hours from HFNC to enrollment	11.1 (4.8–20)	9.4 (4.2–18.6)	–	–
At study enrollment			–	–
Respiratory rate—breaths/min	25.0 ± 4.3	25.3 ± 4.2	–	–
HFNC flow settings—L/min	40 (40–40)	40 (40–40)	–	–
F_I_O_2_	0.7 (0.6–1.0)	0.7 (0.6–1.0)	–	–
SpO_2_/F_I_O_2_	134.7 ± 38.7	135.5 ± 37.9	–	–
ROX index	5.3 (3.7–7.1)	5.5 (3.8–6.9)	–	–
Silent hypoxemia—no. (%) ^b^	58 (27)	59 (27)	–	–
Lung ultrasound score^c^	18 (16–21)	18 (15–22)	–	–
D-dimer—mg/dL	1.2 (0.9–1.6)	1.1 (0.8–1.7)	–	–
Coexisting illness^d^	153 (71)	151 (71)	–	–
Use of glucocorticoids for treatment of COVID-19—no. (%)	182 (84)	184 (86)	–	–
Highest treating location			–	–
Intermediate care unit (patient-to-nurse-ratio 4:1)—no. (%)	172 (80)	162 (76)	–	–
Intensive care unit (patient-to-nurse-ratio 2:1)—no. (%)	44 (20)	52 (24)	–	–
*Outcomes*				
Intubation at day 28—no. (%)	65/216 (30)	92/214 (43)	0.70 (0.54–0.90)	0.006
Mortality at day 28—no. (%)				
All patients	71/216 (33)	79/214 (37)	0.89 (0.68–1.15)	0.37
Patients with IMV	48/65 (74)	59/92 (64)	1.15 (0.93–1.42)	0.18
Treatment success at day 28 (alive without intubation)—no. (%)	128/216 (59)	102/214 (48)	1.28 (1.04–1.57)	0.01
Adverse events				
Skin breakdown—no. (%)	1 (0.5)	3 (1.4)	–	–
Vomiting—no. (%)	5 (2.3)	10 (4.7)	–	–
Intravascular lines dislodgement—no. (%)	14 (6.5)	14 (6.5)	–	–
Back pain—no. (%)	16 (7.4)	13 (6.1)	–	–
Cardiac arrest related to position change	0	0	–	–

			Median difference (95% CI)	
Days of HFNC in patients who had treatment success, median (IQR)	8.7 (7.4–11.7)	9.6 (7.5–12)	− 0.5 (− 1.4–0.2)	0.21
Days from study enrollment to intubation in patients with IMV, median (IQR)	2.8 (1.9–4.1)	2.2 (1.4–3.5)	0.6 (0.1–1.0)	0.02
Days of IMV, median (IQR)	9.4 (6.0–14.5)	10.6 (7.8–14.3)	− 0.9 (− 2.6–0.8)	0.29
Hospital LOS, median (IQR)	11 (9–14)	13 (10–17)	− 1.5 (− 2–0)	0.001

Plus–minus values are means ± SD; median with interquartile ranges is in parentheses. APP, awake prone positioning; HFNC, high-flow nasal cannula; SpO_2_, saturation of pulse oximetry; F_I_O_2_, fraction of inspired oxygen; ROX, SpO_2_/F_I_O_2_ /respiratory rate; CI, confidence interval; RR, relative risk; IMV, invasive mechanical ventilation; LOS, length of stay

^a^Body mass index is the weight in kilograms divided by the square of the height in meters

^b^Silent hypoxemia was defined as SpO_2_ < 90% at ambient air but no perception of symptoms of dyspnea or shortness of breath at hospital admission

^c^Lung ultrasound with 12-lung regions technique, scores range from 0 to 36, with higher scores indicating lower lung aeration

^d^Coexisting illness included: chronic heart disease (known heart failure, coronary artery disease, or hypertension); chronic lung disease (obstructive or restrictive); chronic kidney disease (estimated glomerular filtration rate < 60 mL/min/1.73 m^2^ prior to hospital admission; severe liver disease (cirrhosis and/or portal hypertension with history of variceal bleeding, or liver disease with Child–Pugh score ≥ 10)

**Table 2:** Baseline characteristics and outcomes according to subgroups of silent and dyspneic hypoxemia

	Silent hypoxemia (n = 117)	Dyspneic hypoxemia (n = 313)	RR (95% CI)	*P*
*Characteristics*				
Assigned to APP arm—no. (%)	58 (49.5)	158 (50.5)	–	–
Age, median (IQR)	59 (47–73)	59 (49–71)	–	–
Female sex—no. (%)*	58 (50)	200 (64)	–	–
Body mass index ^a^, median (IQR)	29.0 (27.2–32.4)	28.9 (27.4–32.5)	–	–
Days from symptoms onset to hospital admission, median (IQR)	8 (7–9)	8 (7–10)	–	–
Hours from admission in hospital to enrollment in study, median (IQR)	16 (9–27)	14 (9–24)	–	–
Hours from HFNC to enrollment, median (IQR)	8 (4–20)	9 (5–12)	–	–
Respiratory rate at enrollment, median (IQR)*	25.0 (21.0–27.0)	26.0 (23.0–28.0)	–	–
SpO_2_:FiO_2_ ratio at enrollment, median (IQR)	156 (93–160)	132 (92–160)	–	–
Lung ultrasound score ^b^, median (IQR)*	18 (15–20)	19 (16–22)	–	–
D-dimer mg/dL, median (IQR)	1.1 (0.8–1.4)	1.2 (0.9–1.7)	–	–
Coexisting illness ^c^	87 (74)	217 (69)	–	–
Use of glucocorticoids for treatment of Covid-19—no. (%)	101 (86)	265 (85)	–	–
Highest treating location*			–	–
Intermediate care unit—no. (%)	110 (94)	224 (72)	–	–
Intensive care unit—no. (%)	7 (6)	89 (28)	–	–
*Outcomes*				
Intubation at day 28– no. (%)	29/117 (25)	128/313 (41)	0.60 (0.43–0.85)	0.004
Mortality at day 28– no. (%)				
All patients	27/117 (23)	123/313 (39)	0.58 (0.41–0.84)	0.001
Patients with IMV	21/29 (72)	86/128 (67)	1.07 (0.83–1.39)	0.56
Treatment success at day 28 (alive without intubation)—no. (%)	82/117 (70)	148/313 (47)	1.76 (1.31–2.37)	< 0.001

			Median difference (95% CI)	
Days of HFNC in patients who had treatment success, median (IQR)	9.3 (7.5–12.1)	9.1 (7.4–11.8)	− 0.1 (− 1.0–0.6)	0.60
Days from study enrollment to intubation in patients with IMV, median (IQR)	2.4 (1.9–3.4)	2.4 (1.5–4.1)	0 (− 0.5–0.7)	0.88
Days of IMV, median (IQR)	12 (7.2–14.7)	10 (6.8–13.6)	− 1.1 (− 3.3–1.2)	0.37
Hospital LOS, median (IQR)	12 (9–15)	12 (9–15)	0 (− 1.0–1.0)	0.66

*P ≤ 0.05. Median with interquartile ranges is in parentheses. APP, awake prone positioning; HFNC, high-flow nasal cannula; SpO_2_, saturation of pulse oximetry; F_I_O_2_, fraction of inspired oxygen; ROX, SpO_2_/F_I_O_2_ /respiratory rate; CI, confidence interval; RR, relative risk; IMV, invasive mechanical ventilation; LOS, length of stay. Silent hypoxemia was defined as SpO_2_ < 90% at ambient air but no perception of symptoms of dyspnea or shortness of breath at hospital admission

^a^ Body mass index is the weight in kilograms divided by the square of the height in meters

^b^ Lung ultrasound with 12-lung regions technique, scores range from 0 to 36, with higher scores indicating lower lung aeration

^c^ Coexisting illness included: chronic heart disease (known heart failure, coronary artery disease, or hypertension); chronic lung disease (obstructive or restrictive); chronic kidney disease (estimated glomerular filtration rate < 60 mL/min/1.73 m^2^ prior to hospital admission; severe liver disease (cirrhosis and/or portal hypertension with history of variceal bleeding, or liver disease with Child–Pugh score ≥ 10)

### Outcomes

The primary endpoint of intubation occurred in 92 of 214 (43%) patients assigned to the standard care and in 65 of 216 (30%) patients assigned to APP (relative risk [RR] 0.70; 95% confidence interval [CI_95_] 0.54–0.90, *P* = 0.006) (Table 1). The number-needed-to-treat to avoid one intubation was 8 (CI_95_ 4.5–25.7). NIV was used in 39 (18%) patients in the standard care group and 17 (8%) patients in the APP group, with similar duration from enrollment to NIV initiation (*P* = 0.16). All these patients required intubation.

The median duration from enrollment to intubation was 2.2 days (IQR 1.4–3.5) in patients assigned to standard care and 2.8 days (IQR 1.9–4.1) with APP (*P* = 0.02), with similar duration of invasive ventilation and mortality for intubated patients in both groups. Compared to standard care, the APP group had more treatment success (59% vs 48%, RR 1.28; CI_95_ 1.04–1.57, *P* = 0.01), and a shorter hospital length of stay (11 [IQR 9–14] vs 13 [IQR 10–17] days, *P* = 0.001) (Table 1). Results were similar in *per*-protocol analysis after excluding 16 patients of control group who proned ≥ 1 h (see Additional file 1: Table S1).

Intubation occurred in 29 of 117 (25%) patients with silent hypoxemia and in 128 of 313 (41%) patients with dyspneic hypoxemia (RR 0.60; CI_95_ 0.43–0.85, *P* = 0.004). Mean time from enrollment to intubation was 2.4 days in both groups (*P* = 0.60). Fewer deaths occurred in patients with silent hypoxemia (23%, 27/113) than in patients with dyspneic hypoxemia (39%, 123/313) (RR 0.58; CI_95_ 0.41–0.84, *P* = 0.001) (Table 2).

Among the patients with silent hypoxemia, intubation occurred in 6 of 58 (10%) patients on APP and in 23 of 59 (39%) patients of standard care (RR 0.26; CI_95_ 0.11–0.60, *P* = 0.001) (see Additional file 1: Table S2); for this subgroup, the number-needed-to-treat to avoid one intubation was 3.5 (CI_95_ 2.3–7.2).

### Physiological responses to APP

In the intervention group, the average APP daily duration during HFNC treatment was 9.4 h (IQR, 5.6–12.9) for 6 days (IQR 3.7–9.0). Patients performed an average four sessions/day (IQR 3–5), with an average duration of 3.4 h/session (IQR 3.0–3.6).

**Fig. 2:**
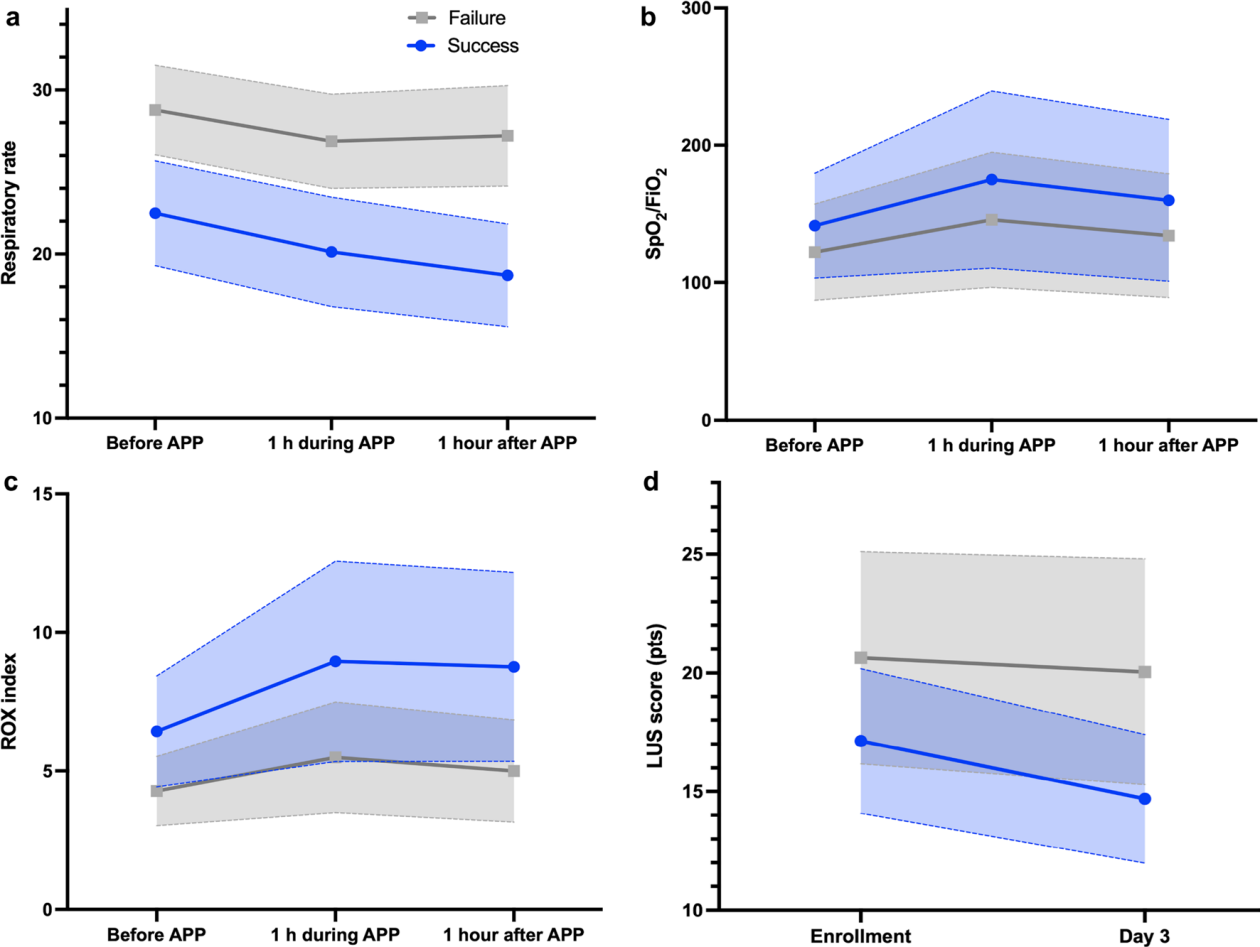
Physiological response to awake prone positioning according to subgroups of treatment success and failure. **a** RR decreased by 1.5 breaths/min in patients with treatment failure vs 3.7 breaths/min in patients with treatment success after the first APP session. **b** SpO_2_/FiO_2_ ratio increased by 12 in patients with treatment failure vs 18 in patients with treatment success after the first APP session. **c** ROX index increased by 0.7 in patients with treatment failure vs 2.3 in patients with treatment success after the first APP session. **d** LUS score decreased 2.4 points in patients with treatment success, while patients with treatment failure had no change after 3 days. RR, respiratory rate; APP, awake prone positioning; LUS, lung ultrasound score

In the first APP session, median respiratory rate decreased from 25 to 22 breaths/min (*P* < 0.001), SpO_2_/F_I_O_2_ increased from 133 to 149 (*P* < 0.001), and the ROX index increased from 5.5 to 7.2 (*P* < 0.001). The physiological response to APP was greater and more sustained in patients with treatment success, with a greater decrease in respiratory rate and increase in SpO_2_/FiO_2_ and in ROX index (Fig. 2a–c).

After 3 days of APP, LUS score decreased from 17.1 (IQR 16.5–17.6) to 14.7 (IQR 14.2–15.1) in patients with treatment success (*P* < 0.001), while patients with treatment failure had no change (Fig. 2d).

### Predictors for treatment success in the overall patient population

In the overall patient population, treatment success was associated with APP, while other variables associated with treatment success included the presence of silent hypoxemia at hospital admission (*P* = 0.01), a lower respiratory rate (*P* < 0.001), higher SpO_2_/F_I_O_2_ (*P* < 0.001), lower lung ultrasound score (*P* < 0.001), and lower D-dimer (*P* < 0.001) at study enrollment (see Additional file 1: Table S3).

### Predictors for treatment success in the APP group

In the APP group, treatment success was associated with longer APP daily duration within the first three days of enrollment (*P* = 0.003). Factors at enrollment associated with APP success included higher ROX index (*P* = 0.009), lower lung ultrasound score (*P* = 0.009), and lower D-dimer (*P* = 0.005). A greater response to APP, including greater improvement in ROX index (*P* < 0.001) and reduction in respiratory rate (*P* < 0.001) after the first APP session and greater reduction in lung ultrasound score at 3 days (*P* = 0.01), was also associated with APP success (see Additional file 1: Table S4).

**Fig. 3:**
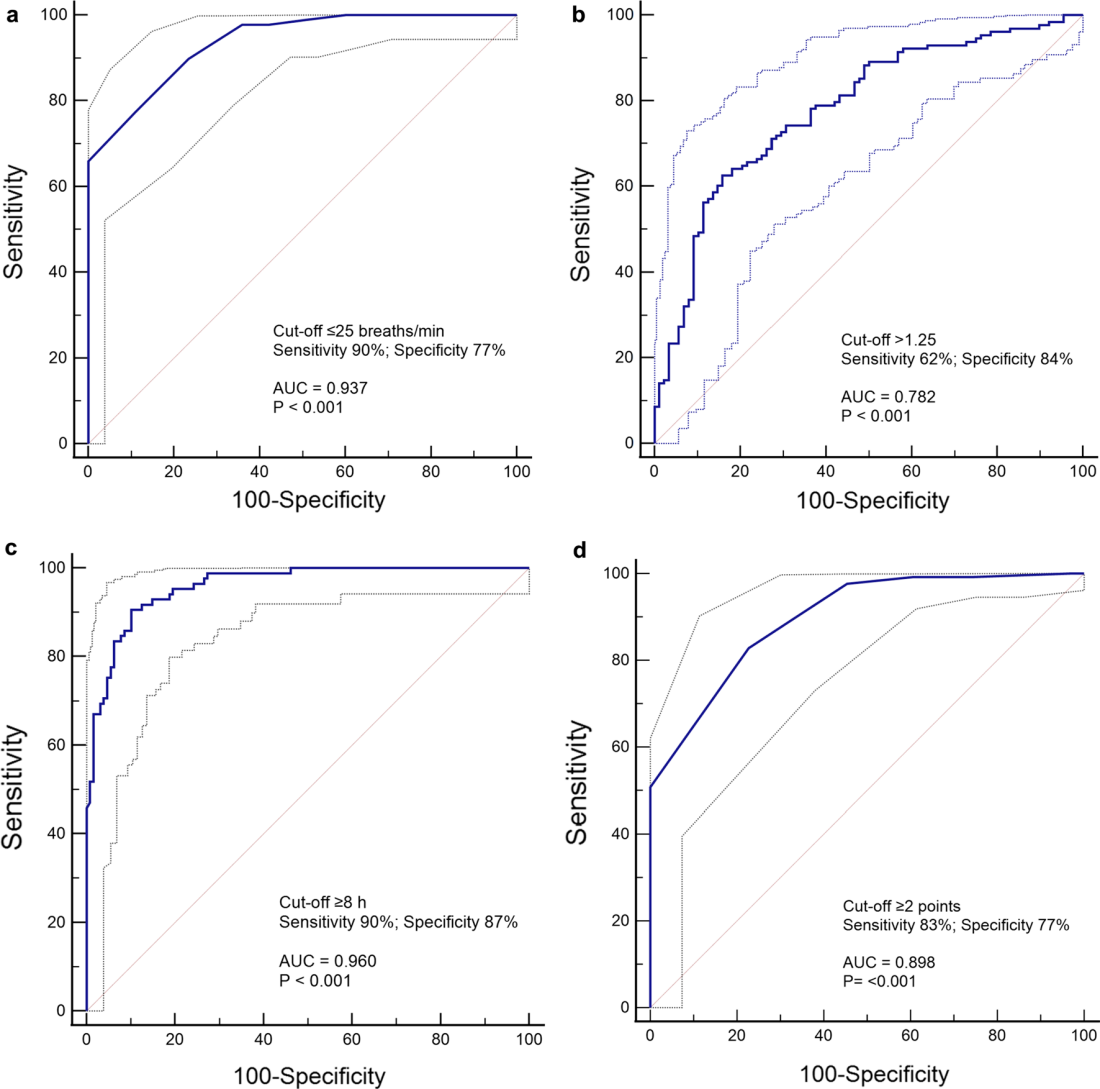
Variables with the highest areas under the curve for prediction of treatment success at day 28 (alive without intubation) in the APP group. **a** Respiratory rate at enrollment. **b** ROX index after the first APP session. **c** Mean daily duration of APP at 3 days. **d** Decrease in LUS score at 3 days. APP, awake prone positioning; LUS, lung ultrasound; AUC, area under the curve

Overall, the cutoff values of variables with the highest areas under the curve for predicting treatment success were: respiratory rate at enrollment ≤ 25 breaths/min (AUC 0.93 [0.90–0.96]), increase in ROX index ≥ 1.25 (AUC 0.78 [0.72–0.83]) after the first APP session, an APP duration > 8 h/day in the first 3 days (AUC 0.96 [0.93–0.98]), and decrease in lung ultrasound score at 3 days ≥ 2 points (AUC 0.73 [0.66–0.80]) (see Additional file 1: Table S5, and Fig. 3).

### APP Duration and outcome

**Fig. 4:**
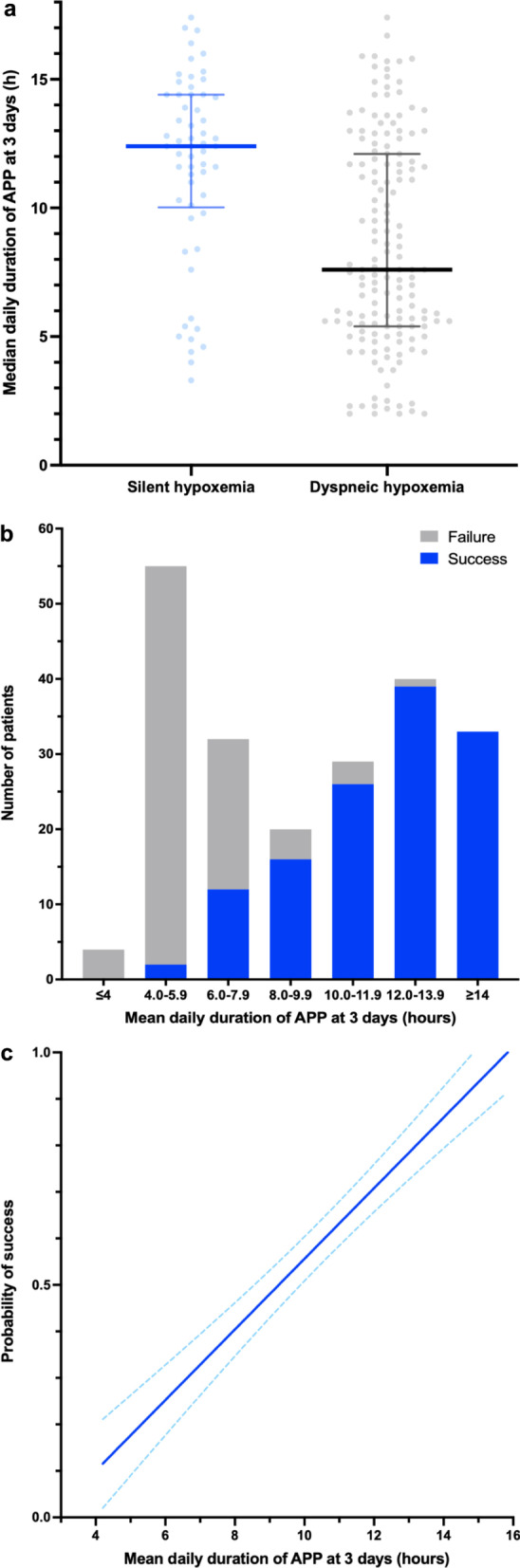
Mean daily duration of APP at the first 3 days. **a** Time on awake prone positioning in patients with silent and dyspneic hypoxemia. **b** Proportion of patients with treatment success rate according to mean daily duration of APP at 3 days. **c** Linear correlation between adjusted probability of failure (according to respiratory rate, SpO_2_/F_I_O_2_, lung ultrasound score, silent hypoxemia, and D-dimer) and mean daily duration of APP at 3 days (r = 0.70, *P* < 0.001) (APP, awake prone positioning)

Patients with silent hypoxemia had a longer time on APP than patients with dyspneic hypoxemia (12.4 [IQR 10.3–14.3] vs 7.6 [IQR 5.4–12.0] hours/day, *P* < 0.001) (Fig. 4a).

**Fig. 5:**
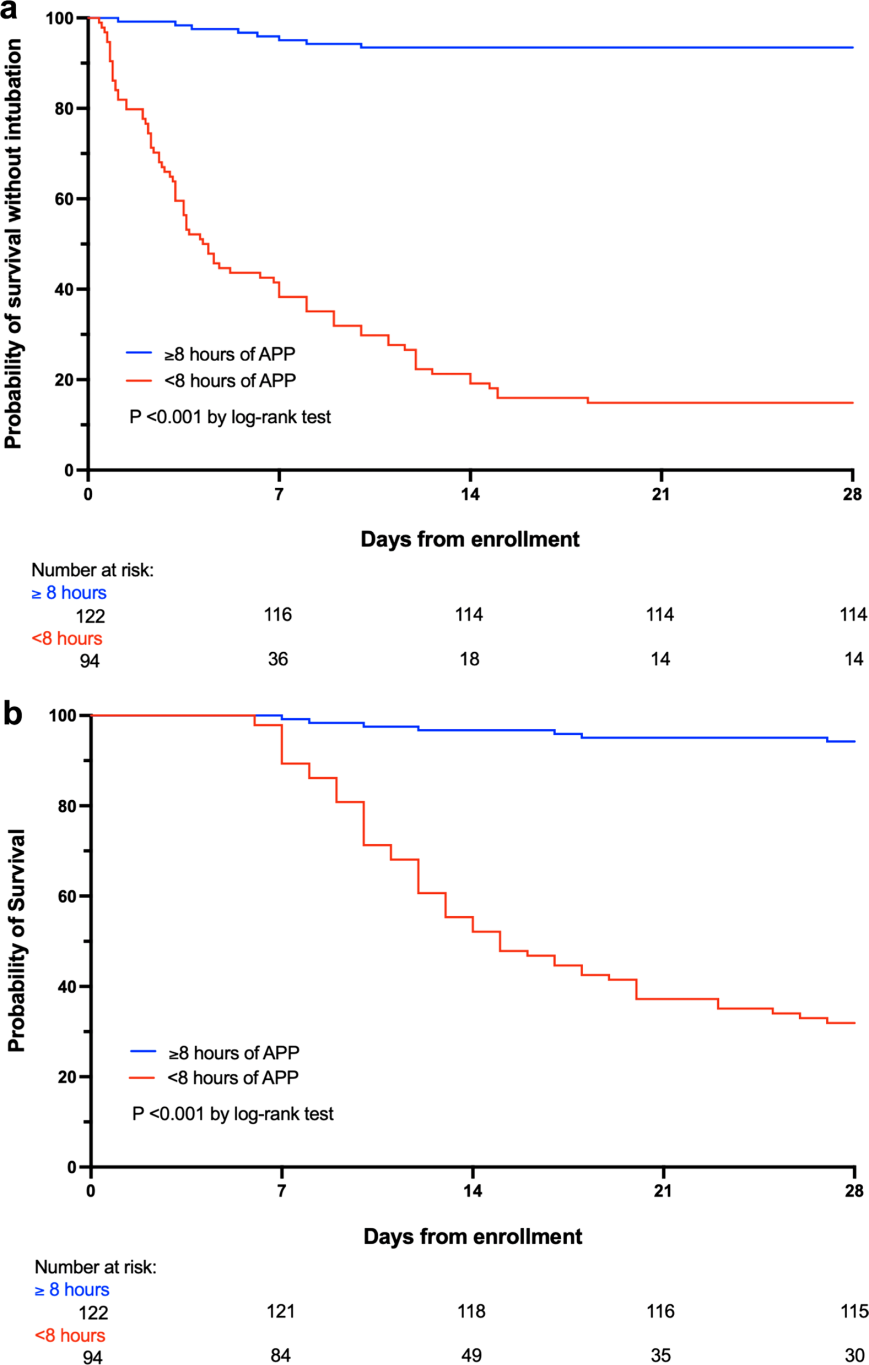
Kaplan–Meier plots of the cumulative incidence of treatment success (**a**) and death (**b**) (APP, awake prone positioning)

Of the 122 patients who had an APP duration in the first 3 days > 8 h/day, 114 (93%) had treatment success; in contrast, for the 94 patients who had daily APP duration < 8 h, only 14 (15%) had treatment success (Fig. 4b). A longer APP duration was significantly correlated with the adjusted risk for treatment success (*r* = 0.70, *P* < 0.001) (Fig. 4c). After adjustment by age, respiratory rate, SpO_2_/F_I_O_2_, lung ultrasound score, D-dimer at enrollment, and silent hypoxemia at hospital admission with Cox proportional hazard regression, patients with APP duration ≥ 8 h/day had an adjusted hazard ratio (HR) of 13.2 (CI_95_ 5.4–32.1) for treatment success (Fig. 5a) and a HR of 5.7 (CI_95_ 2.2–14.5) for survival at 28 days (Fig. 5b) with Kaplan–Meier analysis.

## Discussion

In this multi-center randomized controlled trial, we found that APP was safe and reduced intubation and hospital length of stay in patients with COVID-19-induced AHRF supported by HFNC, with a number-needed-to-treat of eight patients to avoid one intubation. More importantly, the in-depth post hoc analyses revealed predictors of APP success, including an APP duration > 8 h/day, respiratory rate at enrollment ≤ 25 breaths/min , and patient positive response to APP, such as improvement of ROX index and lung ultrasound score after APP.

The APP success in this study can be explained by two main reasons. First, the adherence to the APP protocol by both patients and clinicians was high. Unlike other trials in which patients were only instructed about APP and self-proned at their convenience [19, 20], our APP protocol was driven by healthcare providers, consistent encouragement and in-person assistance were offered, and measures were taken to improve patient tolerance to APP on a 24/7 basis. Second, compared to other trials that investigated the effects of APP [21], our population was more homogeneous regarding severity of disease and level of oxygen support; only HFNC was utilized and protocolized for both groups.

Applying the prone positioning duration that benefits intubated patients to non-intubated patients is very challenging; in fact, only 6% and 27% of patients achieved the ≥ 16 h/day goal in reported studies [21, 22]. Our findings suggest an APP duration ≥ 8 h/day as an effective and attainable target for non-intubated patients. Still, the positive correlation between APP duration and probability of success reinforces the concept that patients should be in the prone position as long as tolerated.

Small observational studies trying to identify baseline predictive factors for intubation and/or death in patients with COVID-19 have been published, including the ROX index [23], *P*/*F* ratio [23, 24], and lung ultrasound score [18]; however, the dynamic response of these and other relevant variables after APP initiation and their association with patient-centered outcomes has not been addressed in large randomized controlled trials. In our study, patients who ultimately had treatment success had a greater reduction in respiratory rate after the first APP session, and their lung ultrasound score at 3 days decreased more when compared to patients with treatment failure. These findings support the hypothesis that APP may halt or decrease the development of patient self-inflicted lung injury, by promoting better lung homogeneity during tidal ventilation in patients on HFNC [25]. Moreover, these results provide several useful predictors for successful APP; in particular, close monitoring of patient response to APP in the first three days can help clinicians early identify patients who are less likely to succeed with APP and need to transition to mechanical ventilation. This agrees with Weiss’s findings among intubated patients with COVID-19, whose response to prone positioning in the subsequent sessions after the first prone positioning session helped identify patients who could survive [26]. Our finding in non-intubated patients reminds clinicians to dynamically assess patient response to APP, avoiding delay in intubation. On the other hand, the predictors can also help allocate resources. Patients who are more likely to succeed might be transported outside ICU to free ICU beds for patients who need close monitoring, which is particularly important in the surge when ICU beds are limited.

The presence of silent hypoxemia does not mean that patients were asymptomatic, as there are many other reported symptoms at hospital presentation since early in the pandemic [27]. However, it has been suggested that silent hypoxemia represents a warning clinical presentation for patients with COVID-19, due to the risk of unexpected rapid decompensation and potential for increased mortality [28]. There are no large datasets based on randomized trials with precisely protocolized care to support this hypothesis. In a retrospective study from Italy, Busana et al. found a hospital mortality of 17.6% and 29.7% in patients with silent and dyspneic hypoxemia, respectively [13]. Similarly, patients with silent hypoxemia in our study had a lower mortality than dyspneic patients. Besides the association of silent hypoxemia with a longer time of APP tolerance in our study, it could be hypothesized that the lower respiratory drive in this subgroup might have decreased their risk of self-inflicted lung injury [29], contributing to better outcomes.

### Limitations

Some limitations should be acknowledged. First, this trial was terminated earlier, slightly missing our updated target sample size. Second, NIV use was more common in patients of control group, and we cannot rule out the potentially worse outcomes associated with its use in our study, as NIV was not protocolized and may have been used as a rescue measure in the sickest patients, which may explain the high failure rate. However, given that a possible benefit of NIV has also been recently reported in a large adaptive randomized controlled trial [30], so any risk of this particular bias should be low. Third, we only included patients requiring HFNC after failure of non-rebreathing mask. Thus, our results may not be generalizable to patients with mild hypoxemia. Fourth, we acknowledge that the lack of blinding may have influenced the decision for intubation despite provision of pre-defined intubation criteria. However, the only signal of bias was a slight delay of intubation in the APP group, which did not affect the duration of invasive ventilation or mortality in intubated patients—this is consistent with a recent meta-analysis in which timing of intubation was not associated with mortality or morbidity in critically ill patients with COVID-19 [31]. Regarding timing of other relevant interventions, it is worth noting that the time from hospital admission to initiation of APP was 17 ± 9.3 h, and more importantly, almost all patients started on APP within 24 h of HFNC initiation (11.1 h [IQR 4.8–20]), which is considered as “early APP” and associated with lower mortality compared to patients who started APP ≥ 24 h after HFNC initiation [32]. These findings support a wait-and-see approach with close monitoring, in which the predictors of treatment success can be used to stratify patients’ risk. Fifth, the maximal APP duration is likely dependent on patients’ motivation, body habitus, and disease severity and could represent not only a dose–response therapeutic relationship, but also a test of fitness, with better outcomes observed in those patients who were able to endure the longest durations, presumably due to a larger physiological reserve. The efficacy threshold demonstrated in this report may not be generalizable to different populations and settings. Finally, these predictors of treatment success must be interpreted cautiously, as they resulted from post hoc analyses and must be confirmed in subsequent large randomized trials.

## Conclusions

In conclusion, APP reduced intubation rate and hospital length of stay among patients with COVID-19-induced AHRF requiring HFNC support, as compared with standard care. A longer daily duration of APP, lower respiratory rate before APP, and positive response to APP in the first three days were associated with more treatment success. Our results support APP use as a standard care applied early and as long as possible, with the goal of minimally 8 h/day.

## Supplementary Information

**Additional file 1**. Explanations for the protocol amendments from the first version. **Table S1.** Outcomes in per-protocol population. **Table S2.** Outcomes in APP and standard care groups in patients with silent hypoxemia only. **Table S3.** Multiple logistic regression for treatment success at day 28 in the overall population. **Table S4.** Multiple logistic regression for treatment success at day 28 in patients on the APP group. **Table S5.** ROC curve analysis of all predictive variables for treatment success at day 28 in the APP group.

## Data Availability

After publication, de-identified data will be available for sharing upon reasonable requests to the corresponding author made by researchers.

## Abbreviations

APP: Awake prone positioning
COVID-19: Coronavirus disease
AHRF: Acute hypoxemic respiratory failure
HFNC: High-flow nasal cannula
RR: Relative risk
IQR: Interquartile range
ARDS: Acute respiratory distress syndrome
RT-PCR: Reverse-transcriptase polymerase chain reaction
SpO_2_: Pulse oximetry oxygen
F_I_O_2_: Fraction of inspired oxygen
NIV: Noninvasive ventilation
WINFOCUS: World Interactive Network Focused on Critical Ultrasound
LUS: Lung ultrasound
ROC: Receiver operating characteristic
HR: Hazard ratio
