# Supplementary material for: Factors for success of awake prone positioning in patients with COVID-19-induced acute hypoxemic respiratory failure: analysis of a randomized controlled trial
Source: Crit Care. 2022 Mar 28;26:84. doi: 10.1186/s13054-022-03950-0 (PMC8958810; doi:10.1186/s13054-022-03950-0)
Supplement: Supplementary file 1 — Additional file 1. Explanations for the protocol amendments from the first version. Table S1. Outcomes in per-protocol population. Table S2. Outcomes in APP and standard care groups in patients with silent hypoxemia only. Table S3. Multiple logistic regression for treatment success at day 28 in the overall population. Table S4. Multiple logistic regression for treatment success at day 28 in patients on the APP group. Table S5. ROC curve analysis of all predictive variables for treatment success at day 28 in the APP group. [file 13054_2022_3950_MOESM1_ESM.docx]

**Supplementary Information**

**Factors for success of awake prone positioning in patients with COVID-19 induced acute hypoxemic respiratory failure: analysis of a randomized controlled trial**

Miguel Ibarra-Estrada* ^1^, MD; Jie Li* ^2^, PhD, RRT; Ivan Pavlov ^3^ MD; Yonatan Perez ^4^, MD; Oriol Roca ^5, 6^, PhD, MD; Elsa Tavernier ^7^, PhD; Bairbre McNicholas ^8^, PhD, MB; David Vines ^2^, PhD, RRT; Miguel Marín-Rosales ^9^, PhD; Alexandra Vargas-Obieta ^1^, MD; Roxana García-Salcido ^10^, MD; Sara A. Aguirre-Díaz ^11^, MD; José A. López-Pulgarín ^1^, MD; Quetzalcóatl Chávez-Peña ^1^, MD; Julio C Mijangos-Méndez ^1^, MD; Guadalupe Aguirre-Avalos ^1^, PhD; Stephan Ehrmann** ^4^, PhD, MD; John G. Laffey** ^8^, MD, MB.

**Contents**

| **Explanations for the protocol amendments from the first version** | **3** |
| --- | --- |
| **Supplementary Table 1**. Outcomes in per-protocol population | **5** |
| **Supplementary Table 2**. Outcomes in APP and standard care groups in patients with silent hypoxemia only | **6** |
| **Supplementary Table 3**. Multiple logistic regression for treatment success at day 28 (being alive without intubation) in the overall population | **7** |
| **Supplementary Table 4.** Multiple logistic regression for treatment success at day 28 (alive without intubation) in patients on the APP group | **8** |
| **Supplementary Table 5.** ROC curve analysis of all predictive variables for treatment success at day 28 (alive without intubation) in the APP group | **9** |
| **References** | **10** |

**Explanations for the protocol amendments from the first version**

**-Recalculation of the sample size**

When we planned the study in the early pandemic using available data [1], we expected an intubation rate of 60% in the standard care group and intubation rate of 40% in the APP group, thus the calculated sample size was 200 patients (100 per group) to provide a statistical power of 80% at an alpha level of 0.05. However, after reaching this sample size, we found the trial was underpowered due to an actual intubation rate of 38% in the standard care group; besides, no boundary for efficacy or futility had been crossed at the second interim analysis of the meta-trial group; therefore, we recalculated the sample size to 468 patients (234 per group) to detect a difference of 12% (38% in the control group versus 26% in the APP group) with the same alpha and beta levels. Institutional review boards approved amendment of the trial on November 6, 2020. No interim analyses were planned other than those performed at the meta-trial level.

**-Added outcomes**

Treatment success

When we planned the study, we did not consider death as a competing event to the primary outcome (intubation), as patients with do-not-intubate/resuscitate order were excluded at screening. However, forty-three (10%) patients changed their mind and died after refusing intubation few days after enrollment, this led us to add “treatment success” as a secondary outcome, defined as being alive without intubation at day 28; considering that those patients could have survived despite not being intubated, and that treatment success is a better reflection of the overall disease progression rather than intubation, we also decided to report secondary analysis based on this outcome.

Use and timing of non-invasive ventilation (NIV)

At an early phase of the pandemic, the use of non-invasive ventilation (NIV) was only considered as an alternative therapy in cases in which HFNC was not available, as there were concerns regarding aerosol transmission and increased risk of infection of healthcare providers [2]. Moreover, there was no compelling evidence on the beneficial role of NIV to reduce intubation or mortality in patients with Covid-19 [3, 4]. It was discouraged according to hospital regulations; therefore, we did not implement its use within the study protocol for patient care; as the Covid-19 situation evolved and transmission risk was found low [5], some clinicians considered NIV as a valid option for escalation in respiratory support, therefore we recorded the use of NIV for enrolled patients.

***-Removed outcomes***

ICU-length of stay

Although the intensive care unit was almost exclusively used for admission of patients with intubation for mechanical ventilation, it also functioned as a high-dependency unit, which led to non-intubated patients being sometimes admitted to the ICU, but then discharged to offer her/his ICU bed to patients with more severe disease. This led to an intense flow of patients between intensive and intermediate care units. Correct attribution of outcomes as occurring specifically during the ICU stay, as opposed to those in the high-dependency units, was not obvious and could have led to incorrect interpretations. We therefore decided not to report any ICU-related outcomes.

**Data already published in the Meta-trial (6)**

As stated in manuscript, this study was part of an already published collaborative meta-trial (6), the following specific variables are already published for patients in both groups: Age, sex, body-mass index, clinical parameters at enrollment (respiratory rate, mean arterial pressure, SpO_2_:FiO_2_ ratio), coexisting illness, confirmed status of COVID-19, use of glucocorticoids for treatment of COVID-19, do-not-intubate order, location at enrollment, duration of prone positioning, and number of intubated and dead patients.

**Supplementary Table 1.** Outcomes in per-protocol population

|  | **Study group** | |  |  |
| --- | --- | --- | --- | --- |
|  | **APP**  **(n = 216)** | **Standard care**  **(n = 198)** | **RR**  **(95% CI)** | **P= value** |
| Intubation– no. (%) | 65/216 (30) | 86/198 (43) | 0.70 (0.54-0.90) | .004 |
| Mortality at day 28– no. (%) |  |  |  |  |
| All patients | 71/216 (33) | 73/198 (37) | 0.89 (0.68-1.15) | .39 |
| Patients with IMV | 48/65 (74) | 54/86 (63) | 1.15 (0.93-1.42) | .15 |
| Treatment success at day 28 (alive without intubation) – no. (%) | 128/216 (59) | 93/198 (47) | 1.30 (1.05-1.60) | .01 |
| Adverse events |  |  |  |  |
| Skin breakdown– no. (%) | 1 (0.5) | 3 (1.5) | 0.33 (0.03-3.1) | .27 |
| Vomiting– no. (%) | 5 (2.3) | 10 (5.1) | 0.49 (0.1-1.4) | .13 |
| Intravascular lines dislodgement– no. (%) | 14 (6.5) | 14 (7.1) | 0.9 (0.4-2.0) | .81 |
| Back pain– no. (%) | 16 (7.4) | 13 (6.6) | 1.2 (0.6-2.4) | .73 |
| Cardiac arrest related to position change | 0 | 0 | - | - |
|  |  |  | Median difference  (95% CI) |  |
| Days of HFNC in patients who had treatment success, median (IQR) | 8.7 (7.4-11.7) | 9.6 (7.5-12.0) | -0.5 (-1.4 – 0.2) | .17 |
| Days from study enrollment to intubation in patients with IMV, median (IQR) | 2.8 (1.9-4.1) | 2.1 (1.4-3.5) | 0.6 (0.1-1.1) | .01 |
| Days of IMV, median (IQR) | 9.4 (6.0-14.5) | 10.6 (7.5-14.8) | -0.9 (-2.6 – 0.9) | .33 |
| Hospital length of stay, median (IQR) | 11 (9-14) | 13 (10-17) | -1.0 (-2 – 0) | .003 |

APP, awake prone positioning; IMV, invasive mechanical ventilation; HFNC, high-flow nasal cannula; IQR, interquartile range; RR, relative risk; LOS, length of stay.

**Supplementary Table 2.** Outcomes in patients with silent hypoxemia according to allocated group

|  | **Study group** | |  |  |
| --- | --- | --- | --- | --- |
|  | **APP**  **(n = 58)** | **Standard care**  **(n = 59)** | **RR**  **(95% CI)** | **P** |
| Intubation at day 28– no. (%) | 6/58 (10) | 23/59 (39) | 0.26 (0.11-0.60) | <0.001 |
| Mortality at day 28– no. (%) |  |  |  |  |
| All patients | 7/58 (12) | 20/59 (34) | 0.35 (0.16-0.77) | 0.005 |
| Patients with IMV | 4/6 (67) | 17/23 (74) | 0.90 (0.48-1.66) | 0.72 |
| Treatment success at day 28 (alive without intubation) – no. (%) | 49/58 (85) | 33/59 (56) | 2.83 (1.45-5.52) | <0.001 |
| Adverse events |  |  |  |  |
| Skin breakdown– no. (%) | 0 | 1 (1.7) | - | - |
| Vomiting– no. (%) | 1 (1.7) | 2 (3.4) | - | - |
| Intravascular lines dislodgement– no. (%) | 6 (10.3) | 4 (6.8) | - | - |
| Back pain– no. (%) | 4 (6.9) | 6 (10.2) | - | - |
| Cardiac arrest related to position change | 0 | 0 | - | - |
|  |  |  | Median difference  (95% CI) |  |
| Days of HFNC in patients who had treatment success, median (IQR) | 9.1 (7.5-12.1) | 9.8 (7.4-11.7) | -0.1 (-1.5 – 1.3) | 0.87 |
| Days from study enrollment to intubation in patients with IMV, median (IQR) | 2.5 (2.0-3.5) | 2.4 (1.8-3.3) | 0.4 (-0.7-1.7) | 0.45 |
| Days of IMV, median (IQR) | 13.5 (7.4-14.7) | 11.5 (7.2-14.7) | 0.5(-4.8 – 6.2) | 0.74 |
| Hospital LOS, median (IQR) | 11 (9-15) | 12 (9-16) | -1 (-2 – 1) | 0.32 |

APP, awake prone positioning; IMV, invasive mechanical ventilation; HFNC, high-flow nasal cannula; IQR, interquartile range; RR, relative risk; LOS, length of stay.

**Supplementary Table 3.** Multiple logistic regression for treatment success at day 28 (being alive without intubation) in the overall population

| **Variable** | **Success**  **(n** **=** **230)** | **Failure**  **(n** **=** **200)** | **Univariate**  **p** | **Multivariate**  **OR (95% CI)** | **Multivariate**  **p** |
| --- | --- | --- | --- | --- | --- |
| APP arm– no. (%) | 128 (56) | 88 (44) | 0.01 | 3.9 (1.66 to 9.12) | 0.001 |
| Age, years, mean ± SD | 56.6 ± 15.7 | 60.3 ± 15.6 | 0.01 | 0.99 (0.97 to 1.02) | 0.79 |
| Respiratory rate at enrollment, bpm, median (IQR) | 22 (20-25) | 28 (27-31) | <0.001 | 0.47 (0.38 to 0.57) | <0.001 |
| SpO_2_/FiO_2_ at enrollment | 156 (103-163) | 116 (91-158) | <0.001 | 1.02 (1.01 to 1.03) | <0.001 |
| Silent hypoxemia– no. (%) | 82 (35.7) | 35 (17.5) | <0.001 | 3.32 (1.25 to 8.80) | 0.01 |
| Lung ultrasound score at enrollment, median (IQR) | 17 (15-19) | 21 (17-24) | <0.001 | 0.72 (0.64 to 0.81) | <0.001 |
| D-dimer, mg/dL, median (IQR) | 1.0 (0.8-1.2) | 1.7 (1.2-2.1) | <0.001 | 0.06 (0.02 to 0.16) | <0.001 |

Goodness-of-fit (Hosmer-Lemeshow) chi-squared = 2.14, P=0.97; AUC, 0.97 (0.95–0.98).

SD, standard deviation; SpO_2_, saturation of pulse oximetry; F_I_O_2_, fraction of inspired oxygen; IQR, interquartile range.

**Supplementary Table 4**. Multiple logistic regression for treatment success at day 28 (alive without intubation) in patients on the APP group

| **Variable** | **Success**  **(n = 128)** | **Failure**  **(n** **=** **88)** | **Univariate**  **p** | **Multivariate**  **OR (95% CI)** | **Multivariate**  **p** |
| --- | --- | --- | --- | --- | --- |
| Mean daily duration of APP– hours, mean (SD) | 12.4 (10.3-14.3) | 5.2 (4.2-6.3) | <0.001 | 2.6 (1.3 to 5.0) | 0.003 |
| Respiratory rate at enrollment– bpm, median (IQR) | 23 (20-25) | 28 (27-31) | <0.001 | - | - |
| Decrease in RR after first session^a^, median (IQR) | 4 (2-5) | 1 (0-3) | <0.001 | - | - |
| SpO_2_/FiO_2_ ratio at enrollment, mean (SD) | 142 ± 38 | 122 ± 35 | <0.001 | - | - |
| Increase in SpO_2_/F_I_O_2_ ratio after first session^a^, median (IQR) | 6 (0-33) | 2 (0-23) | 0.15 | - | - |
| ROX index at enrollment, median (IQR) | 6.3 (4.8-7.9) | 4.0 (3.1-5.4) | <0.001 | 6.0 (1.5-23.7) | 0.009 |
| Increase in ROX after first session^a^, median (IQR) | 1.7 (0.7-3.1) | 0.4 (0.08-1.0) | <0.001 | - | - |
| Silent hypoxemia at enrollment– no. (%) | 49 (38) | 9 (10) | <0.001 | - | - |
| Lung ultrasound score at enrollment, median (IQR) | 17 (15-19) | 21 (17-24) | <0.001 | 0.5 (0.3-0.8) | 0.009 |
| Decrease in lung ultrasound score at day 3, median (IQR) | 3 (2-3) | 0 (-2 – 1) | <0.001 | 4.2 (1.3-13.5) | 0.01 |
| D-dimer at enrollment– mg/dL, median (IQR) | 1.0 (0.8-1.2) | 1.7 (1.2-2.2) | <0.001 | 0.006 (0.00-0.21) | 0.005 |

Goodness-of-fit (Hosmer-Lemeshow) chi-squared= 1.17, P=0.99; AUC, 0.99 (0.97–1.0).

SD, standard deviation; SpO_2_, saturation of pulse oximetry; F_I_O_2_, fraction of inspired oxygen; ROX, SpO_2_/F_I_O_2_/ respiratory rate; IQR, interquartile range.

^a^ Change from pre-APP to 1 h after return to supine. APP denotes awake prone positioning.

Multivariate analyses results are reported for significant variables within the model only.

**Supplementary Table 5**. ROC curve analysis of all predictive variables for treatment success at day 28 (alive without intubation) in the APP group

| **Variable** | **Cut-off** | **AUC**  **(95% CI)** | **p** | **PPV** | **NPV** | **LR+** | **LR-** |
| --- | --- | --- | --- | --- | --- | --- | --- |
| Respiratory rate at enrollment | ≤25 bpm | 0.93 (0.90-0.96) | <0.001 | 91.6 | 72.5 | 7.49 | 0.26 |
| SpO_2_/FiO_2_ ratio at enrollment | >155 | 0.65 (0.58-0.73) | <0.001 | 71.4 | 49.6 | 1.72 | 0.70 |
| ROX index at enrollment | ≥6.0 | 0.81 (0.76-0.87) | <0.001 | 88.0 | 58.6 | 5.02 | 0.48 |
| D-dimer at enrollment | <1.4 mg/dL | 0.82 (0.76-0.88) | <0.001 | 78.8 | 86.2 | 2.56 | 0.11 |
| Lung ultrasound score at enrollment | <19 points | 0.73 (0.66-0.80) | <0.001 | 73.9 | 66.7 | 1.95 | 0.34 |
| Decrease in RR at 1h during first session of APP | ≥2 bpm | 0.59 (0.53-0.66) | 0.01 | 67.3 | 59.1 | 1.42 | 0.48 |
| Increase in SpO_2_/FiO_2_ ratio at 1h during first session of APP | >32 | 0.55 (0.48-0.62) | 0.13 | 70.9 | 47.4 | 1.67 | 0.76 |
| Increase in ROX at 1h during first session of APP | ≥1.61 | 0.71 (0.65-0.77) | <0.001 | 81.5 | 57.3 | 3.03 | 0.51 |
| Decrease in respiratory rate after first session of APP^a^ | ≥3 bpm | 0.79 (0.73-0.84) | <0.001 | 78.3 | 64.6 | 2.49 | 0.38 |
| Increase in SpO_2_/FiO_2_ ratio after first session of APP^a^ | >31 | 0.55 (0.48-0.62) | 0.01 | 75.5 | 45.5 | 2.12 | 0.82 |
| Increase in ROX after first session of APP^a^ | >1.25 | 0.78 (0.72-0.83) | <0.001 | 85.1 | 60.7 | 3.93 | 0.45 |
| Mean daily duration of APP at 3 days | ≥8 h | 0.96 (0.93-0.98) | <0.001 | 93.5 | 85.6 | 9.55 | 0.11 |
| Decrease in lung ultrasound score at 3 days | ≥2 points | 0.89 (0.84-0.93) | <0.001 | 87.6 | 69.9 | 3.64 | 0.22 |

APP, awake prone positioning. PPV: positive predictive value; NPV, negative predictive value; LR+, positive likelihood ratio; LR-, negative likelihood ratio; SD, standard deviation; SpO_2_, saturation of pulse oximetry; F_I_O_2_, fraction of inspired oxygen; ROX, SpO_2_/F_I_O_2_/ respiratory rate.

^a^ Change from pre-APP to 1 h after return to supine.

**References:**

1. Grasselli G, Zangrillo A, Zanella A, Antonelli M, Cabrini L, Castelli A, Cereda D, Coluccello A, Foti G, Fumagalli R, Iotti G, Latronico N, Lorini L, Merler S, Natalini G, Piatti A, Ranieri MV, Scandroglio AM, Storti E, Cecconi M, Pesenti A; COVID-19 Lombardy ICU Network (2020) Baseline Characteristics and Outcomes of 1591 Patients Infected With SARS-CoV-2 Admitted to ICUs of the Lombardy Region, Italy. JAMA 323(16):1574-1581. doi: 10.1001/jama.2020.5394.

2. Alhazzani W, Møller MH, Arabi YM, Loeb M, Gong MN, Fan E, et al. Surviving Sepsis Campaign: guidelines on the management of critically ill adults with Coronavirus Disease 2019 (COVID-19). Intensive Care Med. 2020;46(5):854-87. doi: 10.1007/s00134-020-06022-5.

3. Belenguer-Muncharaz A, Hernández-Garcés H. Failure of non-invasive ventilation after use of high-flow oxygen therapy in patients with SARS-Coronavirus-2 pneumonia. Med Intensiva (Engl Ed). 2020;45(8):e37–9. English, Spanish. doi: 10.1016/j.medin.2020.06.012.

4. Mukhtar A, Lotfy A, Hasanin A, El-Hefnawy I, El Adawy A. Outcome of non-invasive ventilation in COVID-19 critically ill patients: A Retrospective observational Study. Anaesth Crit Care Pain Med. 2020;39(5):579-80. doi: 10.1016/j.accpm.2020.07.012.

5. Gaeckle NT, Lee J, Park Y, Kreykes G, Evans MD, Hogan CJ Jr. Aerosol Generation from the Respiratory Tract with Various Modes of Oxygen Delivery. Am J Respir Crit Care Med. 2020;202(8):1115-1124. doi: 10.1164/rccm.202006-2309OC.

6. Ehrmann S, Li J, Ibarra-Estrada M, Perez Y, Pavlov I, McNicholas B, et al. Awake prone positioning for COVID-19 acute hypoxaemic respiratory failure: a randomised, controlled, multinational, open-label meta-trial. Lancet Respir Med. 2021;9(12):1387-95. doi: 10.1016/S2213-2600(21)00356-8.
